# The Serological Prevalence of Rabies Virus-Neutralizing Antibodies in the Bat Population on the Caribbean Island of Trinidad

**DOI:** 10.3390/v12020178

**Published:** 2020-02-05

**Authors:** Janine F. R. Seetahal, Lauren Greenberg, Panayampalli Subbian Satheshkumar, Manuel J. Sanchez-Vazquez, George Legall, Shamjeet Singh, Vernie Ramkissoon, Tony Schountz, Vincent Munster, Christopher A. L. Oura, Christine V. F. Carrington

**Affiliations:** 1 Department of Preclinical Sciences, Faculty of Medical Sciences, The University of the West Indies, St. Augustine Campus, St. Augustine, Trinidad and Tobago; vernie.ramkisson@sta.uwi.edu (V.R.); christine.carrington@sta.uwi.edu (C.V.F.C.); 2 Poxvirus and Rabies Branch, Centers for Disease Control and Prevention, Atlanta, GA 30329, USA; foe2@cdc.gov (L.G.); xdv3@cdc.gov (P.S.S.); 3 Pan American Food-and-Mouth Disease Centre (PANAFTOSA), Pan American Health Organization, Rio de Janeiro CEP 25045-002, Brazil; sanchezm@paho.org; 4 Faculty of Food Production and Agriculture, The University of the West Indies, St. Augustine Campus, St. Augustine, Trinidad and Tobago; legall.george@gmail.com; 5 School of Pharmacy, Faculty of Medical Sciences, The University of the West Indies, St. Augustine Campus, St. Augustine, Trinidad and Tobago; shamjeet.singh@sta.uwi.edu; 6 Department of Microbiology, Immunology and Pathology, College of Veterinary Medicine and Biomedical Sciences, Colorado State University, Fort Collins, CO 80523, USA; tony.schountz@colostate.edu; 7 Virus Ecology Unit, Laboratory of Virology, Rocky Mountain Laboratories, NIAID/NIH, Hamilton, MT 59840, USA; vincent.munster@nih.gov; 8 School of Veterinary Medicine, Faculty of Medical Sciences, The University of the West Indies, St. Augustine Campus, St. Augustine, Trinidad and Tobago; christopher.oura@sta.uwi.edu

**Keywords:** rabies virus, virus neutralizing antibodies, serology, bats, Trinidad, Caribbean

## Abstract

Rabies virus (RABV) is the only lyssavirus known to be present within the Caribbean. The island of Trinidad, is richly diverse in chiropteran fauna and endemic for bat-transmitted rabies with low RABV isolation rates observed in this population. We aimed to determine the seroprevalence of rabies virus neutralizing antibodies (RVNA) in light of spatio-temporal and bat demographic factors to infer the extent of natural exposure to RABV in the Trinidadian bat population. RVNA titers were determined by the RABV micro-neutralization test on 383 bat samples representing 21 species, comprising 30.9% of local bat diversity, from 31 locations across the island over 5 years. RVNA was positively detected in 33 samples (8.6%) representing 6 bat species (mainly frugivorous) with titers ranging from 0.1 to 19 IU/mL (mean 1.66 IU/mL). The analyses based on a multivariable binomial generalised linear mixed-effects model showed that bat age and year of capture were significant predictors of seropositivity. Thus, juvenile bats were more likely to be seropositive when compared to adults (estimate 1.13; *p* = 0.04) which may suggest early exposure to the RABV with possible implications for viral amplification in this population. Temporal variation in rabies seropositivity, 2012–2014 versus 2015–2017 (estimate 1.07; *p* = 0.03) may have been related to the prevailing rabies epizootic situation. Regarding other factors investigated, RVNA was found in bats from both rural and non-rural areas, as well as in both hematophagous and non-hematophagous bat species. The most common seropositive species, *Artibeus jamaicensis planirostris* is ubiquitous throughout the island which may potentially facilitate human exposure. The findings of this study should be factored into public health assessments on the potential for rabies transmission by non-hematophagous bats in Trinidad.

## 1. Introduction

Rabies is a highly fatal but preventable zoonotic disease of major public health significance [1,2]. The causative agent rabies virus (RABV) is the type species and most ubiquitous of the *Lyssavirus* genus [3]. It is the only lyssavirus known to circulate in the Americas [4]. While, the major global burden of rabies is attributed to dog-mediated transmission [5], sylvatic-transmission is becoming increasing important in the epidemiology of rabies [6,7,8,9]. This is particularly relevant in the Americas with the decline of canine-transmitted cases [10,11,12] and the recognition of distinct RABV variants in numerous bat species [9,13,14]. Due to the aerial nature of their reservoir, these variants are more defined by species than by geographical boundaries [15] and in the Americas, nearly 30 distinct bat RABV variants have been found thus far [16]. Although these variants can be transmitted between bat species and to other mammals [14,17], in Latin America and the Caribbean the vampire bat is the bat species most implicated as a reservoir in this region [18].

The Caribbean island of Trinidad, located just 12 km away from the northeast coast of South America, is richly diverse in chiropteran fauna with 68 identified species, including two species of hematophagous bats [19]. The island is enzootic for bat RABV which has been so far isolated from nine bat species [19,20]. Of these, the hematophagous *Desmodus rotundus* species is considered the most effective vector on account of its feeding practices [21] and to date, is the only bat directly implicated in transmission of the virus on the island [22]. Some earlier unconfirmed studies have suggested that apparently healthy bats could harbour and transmit RABV for extended periods [23,24,25,26], however to date this has not been conclusively proven by modern diagnostic methods. In Trinidad viral isolation from bats has been rare with a rabies positivity proportion of 0.05% (two positive of 4399 tested between 1971 and 2015) obtained from samples acquired mainly through active surveillance in the bat population [27]. Despite the bias towards healthy bats inherent to this type of sampling, it has been suggested that this low proportion is a consequence of virus only being periodically imported from the South American mainland (as bats fly from mainland to island) causing vampire bat epizootics with occasional viral spill-over to the livestock population [22]. Nonetheless, over the last 50 years, despite the apparent low levels of RABV circulation in the bat population, five significant epizootic events have occurred on the island [28].

In light of the apparently low prevalence of virus among bats, rabies antibody levels may be used as an indicator of virus exposure to gauge the risk of virus transmission. Few studies on rabies antibody prevalence have been conducted within the Caribbean [29,30,31,32] and the only report from Trinidad (conducted in 1974 during a small epizootic event) demonstrated a seropositive proportion of 12.8% [29]. We therefore sought to determine the current seroprevalence of rabies virus neutralizing antibodies (RVNA) in the Trinidadian bat population over a period of five years in order to infer the extent of natural exposure to RABVs and the spatio-temporal dynamics of RABV infection in the bat population. We also aimed to determine whether seroprevalence varied with factors related to bat demographics and habitat, with a view to identifying potential risk factors for transmission to susceptible animal and human populations.

## 2. Methods

### 2.1. Bat Trapping and Blood Sample Collection

Bats were caught mainly using mist nets set at ground level at dusk and night from February 2012 to April 2017 on the island of Trinidad (see Table S1). Bat trapping and specimen collection were carried out under special game licenses issued annually by the Wildlife Section, Forestry Division, Ministry of Agriculture, Land and Fisheries, Trinidad and Tobago in accordance with the Government of Trinidad and Tobago Conservation of Wildlife Act (1958) Chapter 67:01 (Section 10). Field and laboratory protocols were approved by the Ethics Committee, Faculty of Medical Sciences, University of the West Indies, St. Augustine Campus (14th February 2012). Bats were transported to the laboratory in individual mesh bags for processing. All bats were apparently healthy at the time of sampling and were sampled only once. Biometrics (i.e., forearm length, weight and head and body length) and the appearance of other external characteristics (e.g., tail, chin warts and nose leaf) were recorded for each animal to aid in their morphological species identification using locally developed field identification keys [19]. Juvenile bats were distinguished from adult bats by the lack of ossification in the epiphyseal joint between the metacarpal and proximal phalanx bones demonstrated upon trans-illumination of the wing. Bats were humanely euthanized by anaesthetic overdose using isoflurane and organ tissues were collected by dissection and stored in-house for other parallel studies and future studies including species identification using genetic methods. Blood was sampled by cardiac puncture and sera was separated by centrifugation and stored at −80 °C until further processing.

### 2.2. Rabies Virus Micro-Neutralization Test

The micro-neutralization test was developed to test smaller volumes of test serum making it an ideal comparison to the traditional rapid fluorescent focus inhibition test (RFFIT) [33]. The micro- neutralization test was conducted following the protocol outlined in Smith et al. [33] using the CVS-11 RABV variant. All serum samples were screened at 1:10 dilution and positive sera were run to end-point. A cut off titer of 1:10 (0.1 IU/mL) was chosen based on the value for 50% neutralization of the challenge virus in accordance with previous studies [33,34].

The procedure for performing the micro-neutralization test involves using four-well Teflon coated slides to perform serial dilutions while employing a humidity chamber throughout the procedure to avoid any evaporation. Minimum essential medium (MEM) (12 µL) was added to each well of each slide and 3 µL of each test serum was serially diluted. Standard rabies immunoglobulin (SRIG) was used as positive control serum in preparing the positive control slide [35]. CVS-11 was prepared for the working dilution at 50 FFD_50_ (50% fluorescent focus forming doses) per mL and 12 µL of this working dilution of virus, was added to each well of each test slides as well as to the positive control slide and the back-titration slide. The slides were incubated in the humidity chamber at 37 °C with 0.5% CO_2_ for 90 min. After completion of this incubation period, 24 µL of mouse neuroblastoma cells (MNA) were added to each well, equivalent to 1.4 × 10^4^ cells per well. Slides were again incubated at 37 °C for 20 h with 0.5% CO_2_ then fixed with acetone and stained with FITC-anti-rabies immunoglobulin (Fujirebio Diagnostics, Malvern, PA, USA) before being observed for the presence of fluorescent foci with a fluorescence microscope. The Reed-Muench method [36] was used to calculate the endpoint titer, which was converted to international units per millilitre (IU/mL) based on comparison to SRIG diluted at 2 IU/mL.

### 2.3. Data and Variables Investigated

RVNA titer values were recorded originally as a continuous variable and they were further categorized as a binary variable, either as positive (≥0.1 IU/mL) or negative (<0.1 IU/mL) serological status. The variables investigated in the study were related to the bat captured, i.e., sex, age and dietary habits; or to external factors such as season of capture (dry vs wet season; i.e., January–May vs June–December), year of capture, urbanization level at capture location, and district of capture.

### 2.4. Statistical Analysis

This investigation aimed to study the factors associated with the rabies serological status of the captured bats. The response variable in the analyses was positive (≥0.1 IU/mL) or negative (<0.1 IU/mL) serological status according to the RVNA titer obtained. Initially, the analysis comprised univariable explorations to investigate the associations between the binary status (i.e., positive or negative) of each bat and the different factors investigated in the study, utilizing a binomial generalized linear model (GLM). The proportion of seropositive bats and the 95% Confidence intervals (CI) were also computed.

The variables significantly associated (*p* < 0.05) in the univariable model were included in a multivariable binomial GLM and a backward elimination process was followed to build the final model. Then, the model evolved, allowing for random effects at the county level, utilizing a binomial generalised linear mixed-effects model (GLMM). This approach allowed accounting for the potential over-dispersion present in our data due to clustering at the level of county. The goodness of fit measurement Akaike’s information criterion (AIC), was used for comparison between nested models. Additionally, substantial changes in the estimated coefficients in the models and increases in standard errors during the model building process were investigated. The Wald tests were used to examine and to present the significance (*p* value < 0.05) of the variables retained in the final model. All the analyses and graphs were performed using the Minitab (version 18) [37] and R statistical software environment [38] using the libraries stats, epicalc and lme4.

## 3. Results

### 3.1. Sample Set

**Figure 1:**
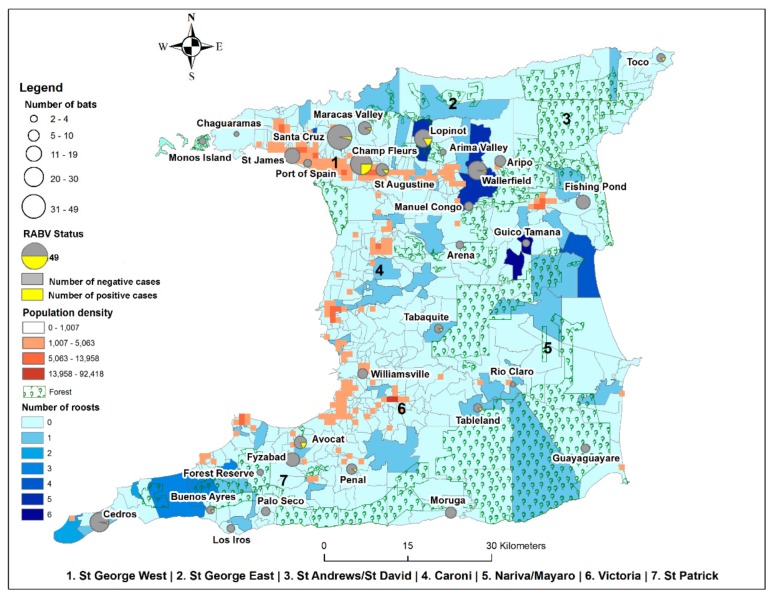
Geographic locations of bat specimen collection in Trinidad relative to human population density, monitored vampire bat roosts and forested areas: numbers of bats collected per site indicated by the size of the circle with the number of RVNA positive (yellow) and negative (grey) samples indicated within each circle; urbanization level indicated by the human population density and; bat roost density indicated by the number of roosts.

In this study, 409 sera samples from bats in Trinidad were collected for RVNA testing indicative of exposure to RABV. Of these, 383 samples representing 31 geographical locations on the island (see Figure 1 and Table S1) were of suitable quality and quantity for determination of RVNA titers.

Twenty-one (21) species of bats representing four of the nine families known to be present on the island, were tested from these locations. The most common species in the sample set was the *Desmodus rotundus* (*n* = 107; 27.9%). After this *Artibeus jamaicensis planirostris* (*n* = 89; 23.2%), *Carollia perspicillata* (*n* = 70; 18.3%) followed by *Molossus molossus* (*n* = 11; 2.9%) bats made up the majority of tested samples.

### 3.2. Bat Serology

RVNA was positively detected in the sera of 33 bats representing 8.6% of the sample population (95% CI 5.81, 11.43) with seropositive titers ranging from 0.1 to 19 IU/mL (mean 1.66 IU/mL; SD ± 4.18), as illustrated in Table S2. Bats from all counties except County Nariva/Mayaro tested positive for rabies antibodies. As detailed in Table 1, County St. George East accounted for the majority of the 33 positive samples (22 of 33; 66.7%). County St. Patrick demonstrated the highest titer values (0.2–19 IU/mL RVNA) in the study (see Table S2). Six species of bats accounted for the 33 seropositive samples (Table S2) and were distributed as follows: sixteen (48.48%) from the species *A. jamicensis planirostris*, six (18.18%) each from *Artibeus lituratus* and *D. rotundus*, three (9.09%) from *C. perspicillata* and one (3.03%) each from *Phyllostomus hastatus* and *Glossophaga soricina*.

### 3.3. Univariate Analyses

#### 3.3.1. Bat Dietary Habits

Most of the seropositive samples (*n* = 33) were from frugivorous (25 of 33; 75.8%) and hematophagous (6 of 33; 18.2%) bat species (Figure S1). Lower numbers of bats with mixed dietary preference and nectarivores were positive when compared to frugivorous and hematophagous species and no insectivorous bats were seropositive for RVNA. Non-hematophagous bats (*n* = 275) demonstrated a seropositivity proportion of 9.8% (95% CI (6.8–13.9); 27 of 275) compared to 5.6% (95% CI (2.6–11.6); 6 of 108) for hematophagous bats (*n* = 108), as illustrated in Table 2. However, there was no significant association between bat diet and seropositivity for rabies antibody (*p* > 0.05) according to the results of the univariable GLM.

#### 3.3.2. Sex and Age

Overall from the entire test population (*n* = 383), more female bats (*n* = 237) were tested than males (*n* = 146). Females accounted for 57.6% (19 of 33) of all positives bats (*n* = 33) as opposed to 42.4% (14 of 33) for males (Figure S1). The seropositivity proportion for males was 9.6% (95% CI (5.3, 15.6)) versus 8.0% (95% CI (5.0, 12.2)) for females. However, there was no significant association between sex and seropositivity for rabies antibody (*p*-value > 0.05) according to the results of the univariable GLM.

Although juvenile bats comprised only 6% (23 of 383) of the test population (*n* = 383) they accounted for 24.2% (8 of 33) of all seropositive samples (see Figure S1). Their seropositivity proportion was 34.8% (95% CI (18.8, 55.1)) significantly higher (*p* ≤ 0.001) than the 6.9% (95% CI (4.8, 10.1)) noted in adult bats. Likewise, there was a statistically significant difference between the mean RVNA titer status in juveniles compared to adults, with juveniles having greater risk (estimate 1.97; *p* < 0.01) according to the results of the univariable GLM). Individual seropositivity status for *Artibeus* juvenile and dam pairs were not consistently similar. For example, in one instance, both the juvenile (T15) and dam (T14) *A. jamaicensis planirostris* were RVNA positive with identical titer values of 0.13 IU/mL, whereas another juvenile bat (T25) of the same species with a similar titer value was associated with a seronegative dam (T24). This dam (T24) was also associated with two other pups (T26 and T27) both of which were seronegative. For the remaining juvenile bats, the dam-pup association was only established for one seronegative adult and juvenile *A. lituratus* pair, which were T37 and T38 respectively. All other juveniles were not affiliated with a dam.

#### 3.3.3. Year and Season of Capture

The majority (81.8%; 27 of 33) of RVNA positive samples were from bats captured during the period 2012–2014 (Figure S1). The seropositivity proportion for this period was 12% (95%CI (8.1, 17.0); 27 of 225) compared to 3.8% (95%CI (1.4, 8.1); 6 of 158) for the period 2015–2017 (Figure S2). The period of bat capture was significantly associated with RVNA serological status, with the first period (2012–2014) having greater risk compared to the second (2015–2017) according to the GLM (estimate 1.24, *p* < 0.01).

About two-thirds of the samples tested (257 of 383) were from bats sampled during the dry season and these accounted for 75.8% (25 of 33) of RVNA positive samples (Figure S1). The prevalence of RVNA positive samples during the dry season (9.7%; 95% CI (6.7, 14.0); 25 of 257) was similar with that for the wet season (6.4%; 95% CI (3.33, 12.0); 8 of 126) with no significant difference in the seropositivity between the seasons (*p* > 0.05) according to the results of the univariable GLM.

#### 3.3.4. Urbanization Level at Capture Location

The majority of tested samples (72.8%; 279 of 383) were from bats trapped in rural areas where there were low human population densities (Figure 1). The prevalence of RVNA positive samples in these areas was 6.5% (95% CI (3.9, 10.0); 18 of 33) compared to 14.4% (95% CI (8.3, 22.7); 15 of 33) in non-rural areas. Seropositive bats from non-rural areas were sampled from both residential and non-residential areas, with 93% (95% CI (70.1–98.9)) of the seropositive samples from non-rural areas sampled at non-residential locations. The association between seropositivity and urbanization level at the location of capture was statistically significant, with urban areas showing a greater risk in comparison to rural areas (estimate 1.42, *p* < 0.01) according to the GLM.

#### 3.3.5. Geographical Location and Method of Capture

Seropositive bats were mainly from two counties, St. George East and St. Patrick, with seropositivity rates of 18.2% (95% CI (11.8, 26.2)) and 6.9% (95% CI (2.6, 14.4)) respectively (Figure S1). Other districts had lower seropositivity rates (2.5–5.9%) and County Nariva/Mayaro had no positives samples (see Table 1). The most seropositive samples per sampled district originated in Champs Fleurs (34.2%; 95% CI (3.9, 10.0); 13 of 38) and Lopinot (20.0%; 95%CI (6.8, 40.7); 5 of 25) in St. George East County and Avocat (23.1%; 95% CI (5.0, 53.5); 3 of 13) in St. Patrick County (Table S2). However, there was no significant difference in the seropositivity across the counties (*p*-value > 0.05) according to the results of the univariable GLM. The seropositivity of bats captured by roost extraction was 9.9% (95% CI (6.2, 14.7); 21 of 213) versus 7.1% (95% CI (3.7, 12.0); 12 of 170) for those captured in the field at the feeding grounds but this difference was not found to be significant (*p* > 0.05) in the GLM.

### 3.4. Multivariable Analysis

The results of the final multivariable GLMM retained two variables as significantly associated with the rabies serological status (see Table 3). Regarding the bat age, the juveniles appeared to have a greater risk that adults and the samples taken during the first period of sampling (2012–2014) also showed a greater risk than those sampled within the second period (2015–2017).

## 4. Discussion

In comparison to viral investigative studies for RABV in bats, there have been limited investigations into the serological prevalence of rabies antibodies in bat populations. In Latin America and the Caribbean, the previous serological studies revealed significantly variable rates for the prevalence of rabies antibodies in bats [29,30,31,39,40,41,42,43,44] which may be attributed to differences in viral population dynamics influenced by spatio-temporal and bat demographic factors as discussed below.

### 4.1. Bat Species

In an earlier Trinidadian rabies seroprevalence study [29], only four of 68 bat species documented on the island (i.e., 5.9% of local bat species diversity) [19] were tested, with only the *Artibeus* species found to be seropositive, compared to 30.9% (21 species) in the present study. In line with studies that show a correlation between the number of bat species from which RABV has been isolated and research effort [13], we found six seropositive bat species and by extension more seropositive bat species and possible isolations of RABV can be expected with more extensive studies covering more bat species. In Trinidad, all except *Phyllostomus hastatus* have previously been reported to be RABV positive [20], while this species was found to be rabies positive in South America [45]. It is important to note that bat taxonomic identification on the basis of morphological characteristics does not distinguish cryptic species so future work will include genetic characterization to clarify species designations.

### 4.2. Rabies Epizootic Situation

Comparison of the seropositivity rate observed in the current study (8.6% for the period 2012–2017) with the rate of 12.8% reported in the 1974 study [29] suggests that there is temporal variation in rabies seropositivity in Trinidad. Rabies serological studies conducted several years apart in French Guiana and Grenada, also showed proportion variations over time, with 6.6% and 10% respectively in earlier years and 10.7% and 7.2% more recently [29,30,31,32]. In our study, year of capture was statistically significantly associated to serological status with, a bat sampled in 2012–2014 having a greater risk of being seropositive than one sampled during 2015-2017. In terms of the rabies epizootic situation at these time, there is evidence of viral exposure during both periods. In 2012, the year in which 79% (26 of 33) of RVNA positive bat samples in our study were sampled (see Table S2), one vampire bat (*n* = 253) was confirmed to be rabies positive [46,47]. On the other hand, in 1974, although no bats were found to be rabid from the small number tested (*n* = 6), 12 bat-transmitted rabies cases were diagnosed in the livestock population clearly indicating virus was circulating in at least the vampire bat population [28]. Although not reported herein, 65 brain samples from bats specimens sampled during the period of this study were tested for RABV and other Lyssaviruses by real-time RT-PCR [48], however none were positive for RABV [49].

### 4.3. Immune Experience

The majority of seropositive samples in this study were <2.0 IU/mL, consistent with natural primary exposure [50]. Some studies have shown that rabies antibody levels decline to undetectable levels between 5 and 6 months post- primary exposure, and up to one year after secondary exposure [50,51]. Similar waning of passive immunity is thought to occur in juvenile bats [50]. So seropositive bats found in this study may have been naturally exposed to RABV up to a year prior to capture which would mean that RABV circulated in the Trinidadian bat population at the very least during the period 2011 to 2016. Only 15% of seropositive bats had RVNA titers higher than 2.0 IU/mL, which could be attributed to immunological priming from past exposures with subsequent anamnestic responses [50,52]. So relatively high rabies antibody titers may occur without active viral infection, because of pre-existing acquired immunity from past exposures [50,53]. This phenomenon may also explain the absence of mass mortalities among bats during epizootic events. Longitudinal temporal monitoring of vampire roosts in Peru and French Guiana [31,41] have provided insights into non-lethal RABV infection by demonstrating seroconversion with highly fluctuating individual seroprevalence rates over time, which perhaps reflects viral persistence in the roost with periodic reactivation. On the contrary, rather than single colony perpetuation of RABV, one study suggests enzootic viral persistence may occur due to movement of infectious bats among colonies, with a high frequency of immunizing non-lethal exposures [54]. Assessment of natural variations in antibody levels for individual bats can be further investigated by capture (mark) and release studies with bat recapture and successive sampling over a period of time to identify changes in RVNA titer levels and determine the maintenance of immunity.

### 4.4. Bat population Density

This study demonstrates variation in rabies seroprevalence by district in Trinidad, with St. George East and St. Patrick accounting for 85% of all RVNA positive samples, consistent with localisation of rabies livestock epizootics in these regions [27,28]. However, more structured sampling across the island, which would account for sample site variation, is necessary to confirm the observed pattern. Differences between regions may be related to the influence of bat population densities on viral dynamics. In theory, large bat roosts provide ideal conditions for viral spread amongst roost mates resulting in larger numbers of bats exposed to higher amounts of virus and thus higher rates of seroconversion [55]. Nevertheless, Streicker et al. [41] found limited evidence for a relationship between vampire bat colony size and exposure to RABV. Affected bats may also be more likely to forgo normal foraging behaviour in preference to staying in roost [56]. Although our results showed no relationship between seropositivity and roost versus field capture, more targeted sampling may reveal underlying associations.

### 4.5. Bat Dietary Habit

The fruit bat, *A. jamaicensis planirostris* was the species most commonly found to be seropositive on the island. This species is highly adaptable and is widespread throughout the island roosting in crevices of both homes and non-residential buildings [19] and therefore has a high potential for human contact. In light of this, public health officials and wildlife biologists should collaborate to address potential risks for human virus exposure with minimum ecological disruption. In general, frugivorous bats had the highest RVNA seroprevalence for Trinidadian bats, which is similar to the situation in Grenada [29,30]. As with other studies, [31,57] RVNA levels varied among bat species (see Table S2) within the same area, suggesting variable rabies exposure and infection dynamics. The wide range of RVNA titer values observed for the hematophagous species (see Table 2) may suggest greater natural exposure to RABV, which may more likely result in abortive infections when compared with other species [53,58]. In the Caribbean (including Trinidad) despite isolation of RABV from 16 bat species, only the *Desmodus* viral variant has thus far been definitely identified [18]. However, the prevalence of RVNA in non-haematophagous bats (particularly *Artibeus* species), in both past and present studies may suggest the presence of other rabies variants or may indicate transmission of the *Desmodus* variant to these species during roost co-habitation [20]. Along these lines, and similar to the situation in other areas [59] non-hematophagous bats may play a significant role in RABV transmission within the Caribbean.

### 4.6. Bat Age

The multivariable GLMM analysis found that juvenile bats were more likely to be seropositive than adults. This is consistent with previous studies which suggest that bats may be exposed to RABV soon after birth [60], and that virus amplification within the susceptible young population can facilitate an increase in RVNA seroprevalence after a birth pulse, through active immunological responses [61,62]. This in turn can facilitate long-term maintenance of the virus [63]. The role of pre-natal exposure is debatable, as contradictory evidence has thus far been provided for in-utero RABV transfer [61,64]. Alternatively, passive transfer of maternal antibodies may have resulted in seropositive juvenile bats, which may not have been naturally exposed to the virus [65]. This may have been the case with the seropositive juvenile and dam pair in this study (T14 and T15). Although, all attempts were made to maintain appropriate identification records for dam-offspring pairs by observational analysis of dam-pup attachment and nursing prior to and after bat capture, alloparental nursing cooperative behaviour [66,67] cannot be ruled out as an explanation for the seropositive juvenile bat paired with a seronegative dam in this study (T25 and T24). Genetic maternity analysis, to confirm the maternal relationships between these adult-juvenile pairs [68], was beyond the scope of the current study, but should be employed in future studies into the relationship between maternal and juvenile RVNA titers. In another study where age was noted to be a significant predictor of RVNA, higher seropositivity rates in juvenile bats compared to adults were thought to reflect active infection rather than the presence of maternally derived antibodies, as titers were much higher in juveniles than the adult females and early antibody decline consistent with passive immunity was not observed [41]. Single timepoint sampling employed in the current study did not allow for assessment of antibody decline, but titer levels were noted to be comparable between the seropositive dam-pup pair. Twelve out of the 33 (36%) seropositive bats identified in this study were from one maternity roost. This finding supports the evidence for a higher viral prevalence in pregnant and lactating female bats [69,70].

### 4.7. RABV Prevalence Rates

The estimated prevalence of RABV in wild bat populations within endemic regions is typically <1% [56,71]. Over the last century, since the isolation of RABV in Trinidadian bats, the RABV prevalence proportion in this population, mainly from active surveillance, has gradually decreased from 3.3% (1930s) to 0.4% (1950-60s) to the most recent estimate of 0.05% (1971–2015) [27,72,73,74]. This apparent decline may reflect lower levels of viral circulation in the bat population and may be responsible for the lower seroprevalence proportion demonstrated in this contemporary study (8.6%), as compared the previous study conducted in the 1970s (12.8%) [29]. Conversely, studies conducted in South America demonstrate an inverse relationship between RABV prevalence and RVNA seroprevalence [43,44,75]. This may be attributed to the protective effect of herd immunity and death of virus-infected individuals, with the virus less able to establish infection and sustain bat to bat transmission due to increasing numbers of immune individuals. Hence it has been suggested that RABV epizootics in bat populations are unidirectionally migratory with a periodicity of at least four years between events, which allows the population to regenerate a threshold of susceptible individuals [76].

### 4.8. Public Health Implications

In Trinidad, bat surveillance has traditionally targeted hematophagous bats with *Desmodus* species comprising the vast majority of tested bats [22,27]. However, the prevalence of RVNA in non-hematophagous bats found in this study suggests an expansion of rabies diagnostic testing to non-hematophagous species may be warranted to obtain a comprehensive picture of rabies host diversity and viral dynamics and ultimately determine the risk of transmission [31]. In general the Trinidadian population is well aware of the risks associated with hematophagous bats due to the history of rabies on the island [27]. However, the potential for rabies transmission by non-hematophagous bats may be overlooked and persons may not seek medical care after exposures to these bats. Although the potential risk for exposure to an actively infected RABV bat is relatively low, our results indicate that the virus can be associated with non-hematophagous bats so adequate precautions must be taken when handling all bats and appropriate post exposure prophylaxis is recommended in accordance with international guidelines regardless of the species of bat [5].

### 4.9. Recommendations and Conclusions

Rabies serological rates from Trinidad and other countries in which rabies is enzootic [40,41,42] were comparable to those found in Grenada [29,30], which is surprising because despite the single historical isolation of RABV from an *Artibeus* bat [29], the virus is not known to be enzootic in the Grenadian bat population. This along with the high RVNA prevalence among the *Artibeus* bats [29,30], which are known to have long distance flight ranges over open water [77] may imply translocation of the virus from endemic areas such as Trinidad by bat movement. Taken together with the ubiquity of the *Artibeus* species throughout the Caribbean [78] the presence or introduction of RABV into other Caribbean islands previously thought to be free of bat rabies is plausible. This has direct implications for the island of Tobago which is only 42 km from Trinidad, compared to 160 km from Trinidad to Grenada. In general, the lack of rabid clinical syndromes in bats may not necessarily reflect the spatial range of lyssaviruses due to exposures resulting in seroconversion rather than overt disease or latent infection [23,79]. Reports of serological evidence of other lyssaviruses in the Old World within areas previously thought to be ‘free’ of these viruses [6,80] have highlighted the value of serological surveillance to determine public health risk. Consequently, serological surveys for anti-RABV antibodies in bat populations within the Caribbean thought to be historically “rabies-free”, especially those islands closer to the American continent, may be worthwhile to establish the true geographical range of RABV in the Americas. Such wide scale investigations might consider prioritizing species demonstrated to have flight ranges over large distances, particularly over open water such as *Artibeus jamaicensis* [77] and *Tadarida brasilensis* [81] species. With respect to sampling effort, surveillance for antibodies may be more efficient than viral detection in the long-term, however, the presence of these antibodies do not necessarily demonstrate active infection, but reflects past exposure to a Phylogroup I Lyssavirus [82]. Therefore viral isolation and typing would be necessary to confirm the Lyssavirus circulating in these bat populations. Similarly in Trinidad, although no other Lyssavirus has been detected here or within the Americas, the detection of RVNA does not necessarily mean that RABV is circulating in the bat population as the existence of a closely related Lyssavirus that elicits a cross-reactive immune response cannot be ruled out [82] Furthermore, seroprevalence levels may vary over time at any given location based on bat population dynamics, infection kinetics and seasonal influences [80], thus caution must be exercised in the extrapolation of prevalence data.

## Figures and Tables

**Table 1:** RVNA prevalence and positive titer values by location (County) of capture.

District of Capture	N Tested (n)	N Positive (a)	RVNA P (%)	95% CI for P	RVNA Positive Titer Value/Range (IU/mL)
Caroni	17	1	5.9	(0.1, 28.7)	0.1
St. George East	121	22	18.2	(11.8, 26.2)	0.1–4.2
St. Andrew/St. David	36	1	2.8	(0.1, 14.5)	0.1
St. Patrick	87	6	6.9	(2.6, 14.4)	0.2–19
St. George West	79	2	2.5	(0.3, 8.8)	0.7–0.17
Nariva/Mayaro	8	0	NA	NA	NA
Victoria	35	1	2.9	(0.1, 14.9)	0.2
**Total**	383	33	8.6	(6.0, 11.9)	0.1–19

NA: not applicable.

**Table 2:** RVNA prevalence and titer values for different bat dietary habits.

Bat Dietary Habit	N Bat Species	N Tested (n)	N Positive (a)	RVNA P (%)	95% CI for P	RVNA Positive Titer Value/Range (IU/mL)
Nectarivorous	3	31	1	3.2	(0.6, 16.2)	0.12
Frugivorous	3	191	25	13.1	(8.7, 18.7)	0.1–4.2
Hematophagous	2	108	6	5.6	(2.1, 11.7)	0.1–19
Insectivorous	6	29	0	NA	NA	NA
Mixed	7	24	1	4.2	(0.7, 20.2)	0.1
Total	21	383	33	8.6	(6.0, 11.9)	0.1–19

**Table 3:** Adjusted estimates and *p*-values for the multivariable binomial GLMM for rabies serological status for bats included in the serological analysis (*n* = 383).

Variable	Estimate	*p*-Value
Sampling period (2012–2014 versus 2015–2017)	1.07	0.03
Bat age (juvenile versus adult)	1.13	0.04

## Supplementary Materials

The following are available online at https://www.mdpi.com/1999-4915/12/2/178/s1, Figure S1: Number of RVNA positive bat samples against total number of bat samples tested by study variable: (i) bat dietary habit; (ii) bat age; (iii) bat gender; (iv) year of capture; (v) season of capture; (vi) human demographics; (vii) district of capture and; (viii) location of capture, Figure S2: Percentage RVNA positive bat samples by season and year of capture, Table S1: Geographic locations and sampled years for the bat serological sample set utilized in the study, Table S2: Demographic and habitat information for RVNA positive bat samples.
