# Supplementary material for: The Serological Prevalence of Rabies Virus-Neutralizing Antibodies in the Bat Population on the Caribbean Island of Trinidad
Source: Viruses. 2020 Feb 5;12(2):178. doi: 10.3390/v12020178 (PMC7077287; doi:10.3390/v12020178)
Supplement: Supplementary file 1 [file viruses-12-00178-s001.zip › viruses-688707-for conversion-suppl_/Supplemental files_proof/Supplementary tables.pdf]

Table S1: Geographic locations and sampled years for bat serological sample set utilized in the study

Location of capture	District of Capture	N tested	N positive	Species of bats (n= number of bats)	Sampled years
Arena	Caroni	5	0	*Carollia perspicillata (3)*	2016, 2017
				Artibeus jamaicensis planirostris (1)	2017
				Uroderma bilobatum (1)	2012
Arima Valley	St. Andrew/ St. David	3	0	*Uroderma bilobatum (1)*	2016
				Artibeus jamaicensis planirostris (2)	2016
Aripo	St. Andrew/ St. David	10	0	*Carollia perspicillata (6)*	2016, 2017
				Artibeus jamaicensis planirostris (2)	2016, 2017
				Pteronotus parnellii (2)	2016, 2017
Avocat	St. Patrick	13	3	*Desmodus rotundus (13)*	2012
Buenos Ayres	St. Patrick	5	1	*Desmodus rotundus (5)*	2012
Cedros	St. Patrick	30	1	*Desmodus rotundus (1)*	2015
				Carollia perspicillata (29)	2017
Chagaramas	St. George West	2	0	*Desmodus rotundus (2)*	2017
Champs Fleurs	St. George East	38	13	*Artibeus jamaicensis planirostris (32)*	2012, 2017
				Phyllostomus discolor (4)	2017
				Sturnira tildae (1)	2017
				Carollia perspicillata (1)	2013
Fishing pond	St. Andrew/ St. David	16	0	*Glossophaga soricina (13)*	2017
				Glossophaga longirostris (3)	2017
Forest Reserve	St. Patrick	3		*Desmodus rotundus (3)*	2016
Fyzabad	St. Patrick	16		*Desmodus rotundus (16)*	2012, 2016, 2017
Guayaguayarare	Nariva/ Mayaro	6		*Desmodus rotundus (6)*	2013
Guico Tamana	St. Andrew/ St. David	4	0	*Glossophaga soricina (2)*	2016, 2017
				Artibeus jamaicensis planirostris (1)	2017
				Carollia perspicillata (1)	2017

Location of capture	District of Capture	N tested	N positive	Species of bats (n= number of bats)	Sampled years
Lopinot	St. George East	25	5	*Artibeus jamaicensis planirostris (4)*	2012
				Artibeus lituratus (15)	2012
				Glossophaga soricina (2)	2012
				Saccopteryx bilineata (1)	2012
				Sturnira lilium (3)	2012
Los Iros	St. Patrick	5	0	*Desmodus rotundus (2)*	2016
				Artibeus jamaicensis planirostris (3)	2016
Manuel Congo	Caroni	6	0	*Sturnira lilium (1)*	2017
				Platyrrhinus fusciventris (1)	2017
				Carollia perspicillata (1)	2017
				Glossophaga soricina (1)	2017
				Artibeus jamaicensis planirostris (2)	2017
Maracas Valley	St. George East	14	1	*Carollia perspicillata (2)*	2012
				Artibeus jamaicensis planirostris (1)	2012
				Artibeus lituratus (2)	2012
				Molossus molossus (9)	2016
Monos Island	St. George West	4	0	*Diaemus youngi (1)*	2017
				Noctilio leporinus (3)	2017
Moruga	Victoria	10	0	*Desmodus rotundus (3)*	2016
				Lonchorhina aurita (1)	2016
				Uroderma bilobatum (2)	2016
				Artibeus jamaicensis planirostris (4)	2016
Palo Seco	St. Patrick	6	0	*Desmodus rotundus (6)*	2016
Penal	St. Patrick	9	1	*Desmodus rotundus (9)*	2016
Port of Spain	St. George West	5	0	*Artibeus jamaicensis planirostris (3)*	2016
				Artibeus lituratus (2)	2016
Rio Claro	Nariva/ Mayaro	2	0	*Molossus rufus (2)*	2016

Location of capture	District of Capture	N tested	N positive	Species of bats (n= number of bats)	Sampled years
Santa Cruz	St. George West	49	2	*Artibeus lituratus (13)*	2012
				Saccopteryx bilineata (3)	2012
				Artibeus jamaicensis planirostris (4)	2012
				Carollia perspicillata (16)	2012, 2016, 2017
				Sturnira tildae (1)	2016
				Glossophaga soricina (1)	2016
				Desmodus rotundus (7)	2013, 2016
				Uroderma bilobatum (3)	2016
				Pteronotus parnellii (1)	2016
St. Augustine	St. George East	13	2	*Molossus molossus (2)*	2016
				Glossophaga soricina (6)	2017
				Artibeus jamaicensis planirostris (5)	2016, 2017
St. James	St. George West	19	0	*Artibeus jamaicensis planirostris (19)*	2012, 2016
Tabaquite	Caroni	6	1	*Anoura geoffroyi (2)*	2016
				Phyllostomus hastatus (3)	2016
				Carollia perspicillata (1)	2016
Tableland	Victoria	17	1	*Desmodus rotundus (17)*	2016
Toco	St. Andrew/ St. David	6	1	*Artibeus jamaicensis planirostris (6)*	2016, 2017
Wallerfield	St. George East	28	1	*Desmodus rotundus (17)*	2012, 2016
				Pteronotus parnellii (5)	2016
				Mormoops megalophylla (3)	2016
				Glossophaga soricina (1)	2016
				Carollia perspicillata (2)	2016
Williamsville	Victoria	8	0	*Carollia perspicillata (8)*	2016
**Total**		**383**	**33**		

Table S2: Demographic and habitat information for RVNA positive bat samples

Location-District	No. bats tested (RVNA positive)	Bat ID	Year	Season	Location-County	Urbanization Level	Method of capture	RVNA positive bat species	Feeding behavior	Sex	Age	RVNA titer level (IU/ml)
Avocat	13 (3)	T135	2012	WS	St. Patrick	Ru	Field	*Desmodus rotundus*	Sanguivore	F	A	0.3
		T136	2012	WS	St. Patrick	Ru	Field	*Desmodus rotundus*	Sanguivore	M	A	19
		T143	2012	WS	St. Patrick	Ru	Field	*Desmodus rotundus*	Sanguivore	M	A	15.6
Buenos Ayres	5 (1)	T166	2012	WS	St. Patrick	Ru	Field	*Desmodus rotundus*	Sanguivore	M	A	0.2
Cedros	30 (1)	T370	2017	DS	St. Patrick	Ru	Roost	*Carollia perspicillata*	Frugivore	F	A	0.31
Champs Fleurs	38 (13)	T3	2012	DS	St. George East	NRu-nr	Roost	*Artibeus jamaicensis planirostris*	Frugivore	F	A	0.13
		T5	2012	DS	St. George East	NRu-nr	Roost	*Artibeus jamaicensis planirostris*	Frugivore	F	A	0.11
		T8	2012	DS	St. George East	NRu-nr	Roost	*Artibeus jamaicensis planirostris*	Frugivore	F	A	0.13
		T9	2012	DS	St. George East	NRu-nr	Roost	*Artibeus jamaicensis planirostris*	Frugivore	F	A	3.1
		T14	2012	DS	St. George East	NRu-nr	Roost	*Artibeus jamaicensis planirostris*	Frugivore	F	A	0.13
		T15	2012	DS	St. George East	NRu-nr	Roost	*Artibeus jamaicensis planirostris*	Frugivore	M	J	0.13
		T17	2012	DS	St. George East	NRu-nr	Roost	*Artibeus jamaicensis planirostris*	Frugivore	M	A	0.13
		T18	2012	DS	St. George East	NRu-nr	Roost	*Artibeus jamaicensis planirostris*	Frugivore	F	A	0.62
		T19	2012	DS	St. George East	NRu-nr	Roost	*Artibeus jamaicensis planirostris*	Frugivore	M	J	0.84
		T22	2012	DS	St. George East	NRu-nr	Roost	*Artibeus jamaicensis planirostris*	Frugivore	M	J	0.31
		T23	2012	DS	St. George East	NRu-nr	Roost	*Artibeus jamaicensis planirostris*	Frugivore	F	J	2.0
		T25	2012	DS	St. George East	NRu-nr	Roost	*Artibeus jamaicensis planirostris*	Frugivore	F	J	3.4
		T345	2017	DS	St. George East	Ru	Field	*Artibeus jamaicensis planirostris*	Frugivore	F	A	0.12
Lopinot	25 (5)	T32	2012	DS	St. George East	Ru	Roost	*Artibeus lituratus*	Frugivore	M	J	0.13
		T34	2012	DS	St. George East	Ru	Roost	*Artibeus lituratus*	Frugivore	F	A	0.13
		T36	2012	DS	St. George East	Ru	Roost	*Artibeus lituratus*	Frugivore	F	J	0.13
		T39	2012	DS	St. George East	Ru	Roost	*Artibeus lituratus*	Frugivore	F	J	0.6
		T42	2012	DS	St. George East	Ru	Roost	*Glossophaga soricina*	Nectarivore	F	A	0.12
Maracas Valley	14 (1)	T81	2012	DS	St. George East	Ru	Field	*Artibeus lituratus*	Frugivore	M	A	4.2
Penal	9 (1)	T188	2012	WS	St. Patrick	Ru	Field	*Desmodus rotundus*	Sanguivore	M	A	1.2
Santa Cruz	49 (2)	T171	2012	DS	St. George West	NRu-r	Roost	*Artibeus lituratus*	Frugivore	M	A	0.17
		T107	2012	WS	St. George West	Ru	Field	*Carollia perspicillata*	Frugivore	M	A	0.7

Location-District	No. bats tested (RVNA positive)	Bat ID	Year	Season	Location-County	Urbanization Level	Method of capture	RVNA positive bat species	Feeding behavior	Sex	Age	RVNA titer level (IU/ml)
St. Augustine	13 (2)	T337	2017	DS	St. George East	NRu-nr	Field	*Artibeus jamaicensis planirostris*	Frugivore	M	A	0.2
		JC347	2017	DS	St. George East	NRu-nr	Field	*Artibeus jamaicensis planirostris*	Frugivore	F	A	0.15
Tabaquite	6 (1)	T293	2016	DS	Caroni	Ru	Roost	*Phyllostomus hastatus*	Mixed	M	A	0.1
Tableland	17 (1)	T150	2012	WS	Victoria	Ru	Field	*Desmodus rotundus*	Sanguivore	F	A	0.2
Toco	6 (1)	JC225	2016	DS	St. Andrew/ St. David	Ru	Roost	*Artibeus jamaicensis planirostris*	Frugivore	F	A	0.1
Wallerfield	28 (1)	T182	2012	WS	St. George East	Ru	Field	*Carollia perspicillata*	Frugivore	F	A	0.1

DS: Dry season
WS: Wet season
Ru: rural
NRu-nr: non-rural, non-residential
NRu-r: non-rural, residential
