# Supplementary figures and images for: The Serological Prevalence of Rabies Virus-Neutralizing Antibodies in the Bat Population on the Caribbean Island of Trinidad
Source: Viruses. 2020 Feb 5;12(2):178. doi: 10.3390/v12020178 (PMC7077287; doi:10.3390/v12020178)

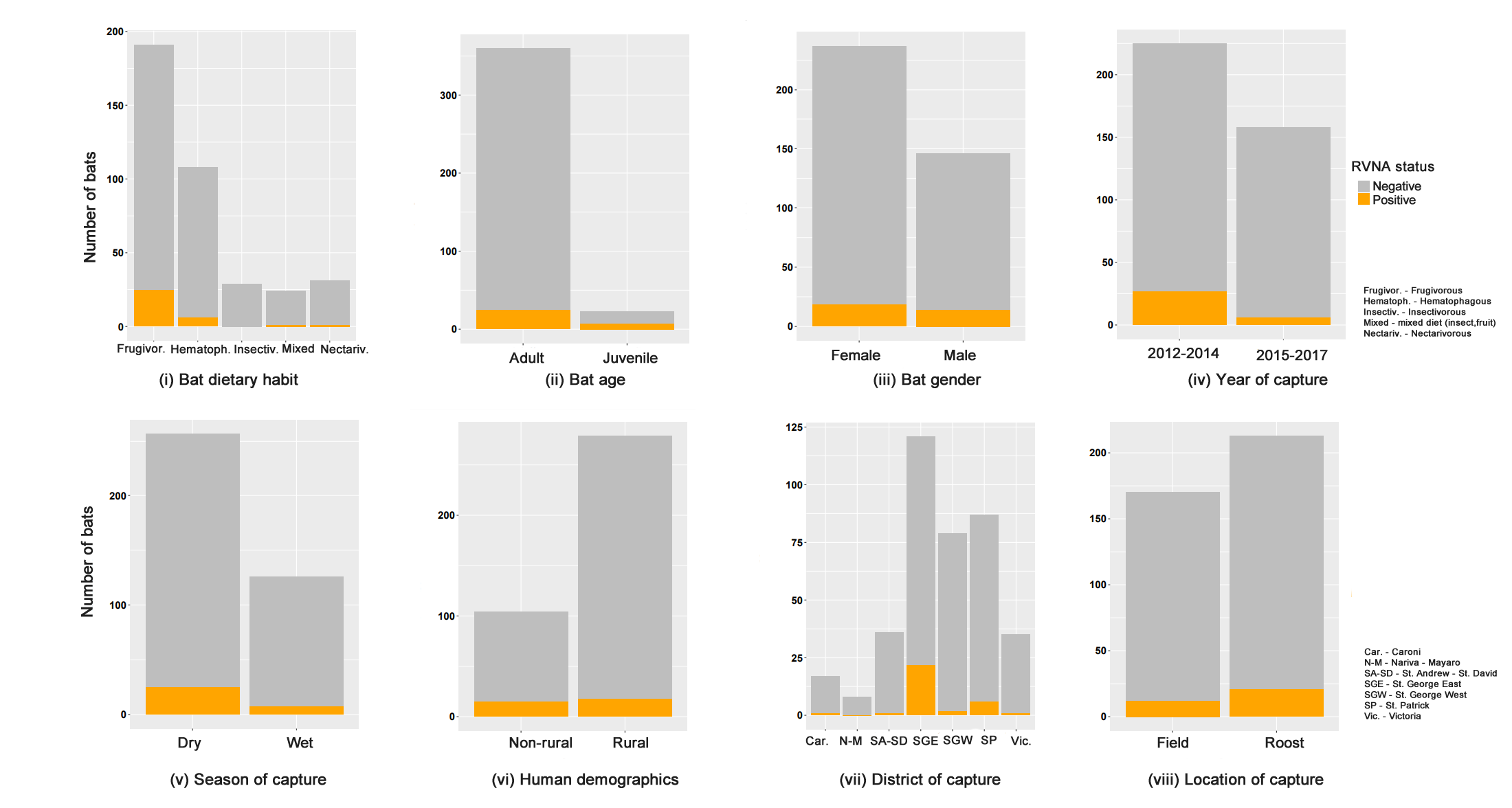

Supplement: Supplementary file 1 [file viruses-12-00178-s001.zip › viruses-688707-for conversion-suppl_/Supplemental files_proof/S1 Figure_Graphs illustrating pos to total numbers.fw.png]

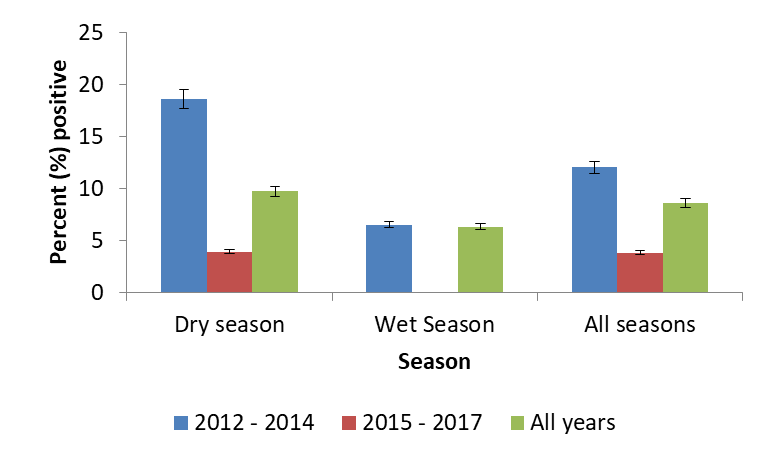

Supplement: Supplementary file 1 [file viruses-12-00178-s001.zip › viruses-688707-for conversion-suppl_/Supplemental files_proof/S2 Figure_percentage pos by season and year.fw.png]
